# Local Diagnostic Reference Levels for Paediatric Head CT Procedures

**DOI:** 10.15388/Amed.2021.28.2.13

**Published:** 2021-08-31

**Authors:** Birutė Gricienė, Monika Šiukšterytė

**Affiliations:** Faculty of Medicine, Vilnius University, Vilnius, Lithuania Vilnius University Hospital Santaros Klinikos, Vilnius, Lithuania ORCID: https://orcid.org/0000-0002-9224-6512; Faculty of Medicine, Vilnius University, Vilnius, Lithuania

**Keywords:** paediatric, computed tomography, diagnostic reference levels, patient exposure, ionising radiation

## Abstract

**Background.:**

Patients, especially children, are exposed to substantially high doses of ionising radiation during computed tomography (CT) procedures. Children are several times more susceptible to ionising radiation than adults. Diagnostic reference levels (DRLs) are an important tool for monitoring and optimising patient radiation exposure from radiological procedures. The aim of this study is to estimate the ionising radiation exposure doses and set local DRLs for head CT examinations according to age and to compare local DRLs with national and European DRLs and with literature data in other countries.

**Materials and methods.:**

Scan parameters of single-phase head CT examinations were collected. Patients were grouped by age in the following intervals: <1, 1−5, 5−10, 10−15 and 15−18 years. Local age-based DRLs set as the 3^rd^ quartile of the median dose-length product (DLP) were calculated. Literature analysis was performed on *PubMed* search engine on inclusion criteria: publication date 2015–2020, used keywords *paediatric computed tomography, paediatric CT, diagnostic reference levels (DRLs)*. The 23 articles discussing paediatric DRLs were further analysed.

**Results.:**

Data was collected from 194 paediatric head CT examinations performed in 2019. The median DLP values for head CT were 144.3, 233.7, 246.4, 288.9, 315.5 for <1, 1−5, 5−10, 10−15 and 15−18 years old groups. Estimated local DRLs for head CT examinations are 170, 300, 310, 320, 360 mGy*cm for <1, 1−5, 5−10, 10−15 and 15−18 years age groups respectively and 130, 210, 275, 320 mGy*cm for 0−3 months, 3 months−1 year, 1−6 years and ≥ 6 years age groups respectively.

**Conclusions.:**

Results of this study showed that settled new local DRLs of head CT examinations were 2–4 times lower than national DRLs and about 2 times lower than European DRLs. Moreover, the study indicated that paediatric head CT doses are significantly lower in comparison with those indicated in the majority of published data from other hospitals over the last 6 years. Patient dose assessment and local DRLs establishment plays important role in future exposure optimisation.

## Abstract (lt)

**Vietiniai diagnostiniai atskaitos lygiai vaikų galvos KT tyrimams:**

**Santrauka. *****Įvadas*****. **Kompiuterinės tomografijos (KT) procedūrų metu pacientai, ypač vaikai, gauna dideles jonizuojančiosios spinduliuotės dozes. Vaikai yra kelis kartus jautresni jonizuojančiajai spinduliuotei nei suaugusieji. Diagnostiniai atskaitos lygiai (DAL) yra svarbi priemonė paciento apšvitai, gautai radiologinių procedūrų metu, vertinti ir optimizuoti. Šio tyrimo tikslas yra įvertinti vaikų galvos KT apšvitą, nustatyti vietinius DAL vaikų galvos KT tyrimams ir palyginti juos su nacionaliniais ir europiniais DAL bei kitų šalių vietiniais DAL vaikų galvos KT tyrimams.

***Medžiaga ir metodai. ***Buvo surinkti vienos fazės galvos KT tyrimų parametrai. Pacientai sugrupuoti pagal amžių (<1, 1−5, 5−10, 10−15 ir 15−18 m.) ir apskaičiuotos vietinių DAL vertės. Literatūros paieška buvo atlikta „PubMed“ duomenų bazėje pagal šiuos kriterijus: publikacijos data 2015–2020 m., vartojami raktiniai žodžiai *vaikų kompiuterinė tomografija, vaikų KT, diagnostiniai atskaitos lygiai, DAL*. Analizei buvo pasirinkti 23 straipsniai, kuriuose aptariami vaikų galvos KT tyrimų DAL.

***Rezultatai. ***Duomenys buvo surinkti iš 194 vaikų galvos KT tyrimų, atliktų 2019 metais. Dozės ir ilgio sandaugos (DLP) medianos vertės buvo 144,3, 233,7, 246,4, 288,9, 315,5 atitinkamai <1, 1−5, 5−10, 10−15 ir 15−18 amžiaus grupėms. Nustatyti galvos KT vietiniai DAL (170, 300, 310, 320, 360 mGy*cm atitinkamai <1, 1−5, 5−10, 10−15 ir 15−18 amžiaus grupėms ir 130, 210, 275, 320 mGy*cm atitinkamai 0−3 mėn., 3 mėn.−1 m., 1−6 m. ir ≥ 6 m. amžiaus grupėms).

***Išvados. ***Šio tyrimo rezultatai parodė, kad mūsų nustatyti vietiniai DAL vaikų galvos KT tyrimams yra 2–4 kartus mažesni nei nacionaliniai šalies DAL ir apie 2 kartus mažesni nei europiniai DAL. Be to, tyrime įvertintos vaikų galvos KT apšvitos dozės buvo mažesnės, palyginti su dauguma publikuotų ligoninių duomenų per paskutinius 6 metus.

Literatūros analizė rodo, kad yra mažas publikacijų skaičius šia tema Europos lygmeniu, todėl svarbu tęsti vaikų KT procedūrų apšvitos dozių optimizavimo tyrimus.

**Raktiniai žodžiai: **vaikai, kompiuterinė tomografija, diagnostiniai atskaitos lygiai, pacientų apšvita, jonizuojančioji spinduliuotė

## Introduction

Computed tomography (CT) is the imaging technique that creates two-dimensional cross-sectional images from three-dimensional body structures and allows physicians to examine the human bones, blood vessels and soft tissues one slice at a time [1-3]. CT procedures are the main source of medical radiation exposure accounting for 24–34% of the world’s annual collective dose, and its use in healthcare is rapidly expanding during the last years [3, 4].

CT scan is a lifesaving and medically beneficial diagnostic tool, however, it creates at least 5–20 times higher ionizing radiation levels than conventional radiographic techniques [4-6]. The patients are exposed to substantially high doses of radiation during CT procedures – a chest CT delivers 100 times the chest X-ray radiation [1, 5]. In addition to this, three CT procedures are performed for 30% of patients, five CT procedures are performed for 7% of patients, and 4% of patients are scanned 9 times and more in a lifetime [1]. Approximately 3–11% of CT scans are performed in the population below 21 years old [7]. The most scanned anatomical regions in children and adolescents are the head [2, 4, 6, 7], the thorax and the abdomen [4, 6]. CT is a standard diagnostic method for detecting paediatric cancer, appendicitis, heart diseases, renal calculi and traumas and is a very useful tool allowing to carry out the procedure without sedation for children [1].

Despite this imaging technique’s speed and accuracy, the excess use of paediatric CT is associated with some risks that must be considered. Ionizing radiation exposure can cause two types of detrimental biological effects – deterministic and stochastic ones. Deterministic effects occur when the threshold high radiation dose is exceeded over a short period. High doses result in severe outcomes, for instance, radiation-induced cataract, skin erythema, sterility, and hair loss. Stochastic effects are caused by series of events that provoke cell transformation and mutation and lead to cancer induction. There is no threshold dose, and it is generally assumed that the risk increases proportionally with dose [1].

Children are 2–10 times more susceptible to ionizing radiation than adults depending on children’s age [1, 7]. They get relatively higher effective doses because of their higher radiosensitivity. In addition to this, children have a longer lifespan after radiation exposure compared to adults and are also more likely to undergo repeated CT scans throughout their lifetime [1, 6, 8]. The most exposed to ionizing radiation are organs and tissues such as red bone marrow, brain, thyroid gland, etc. [3]. Leukaemia and intracranial tumors are the most common cancers in childhood [10]. The risk of central nervous system cancer and leukaemia is increased concerning cumulative CT radiation dose to the brain and the red bone marrow [2, 3, 6, 9-11]. The cumulative dose of 2–3 head CTs triples the risk of leukaemia and also might triple the risk of brain cancer [10]. Though the individual risk of cancer over life is relatively small, the increased numbers of CT procedures have become a public health issue. In the ten years after the first CT scan for patients younger than 10 years old, one case of brain cancer and one case of leukaemia per 10000 CT scans are estimated to occur [10]. There is also a risk of other solid cancers, e.g., breast [6] and thyroid cancers [3, 6]. Thyroid cancer induction is most common from head and neck CTs performed during childhood [7]. There is a stronger risk of developing thyroid cancer in women and leukaemia in men [3]. A limited number of publications found a positive association between radiation dose from CT scans and the incidence of lymphomas [2, 3]. 

Therefore, it is reasonable to act on the assumption that there may be some cancer risk, and the goal to reduce unnecessary medical radiation exposure in paediatric patients remains. Justification and radiation protection optimization are the main principles for reducing radiation exposure from CT scans [5, 6]. According to the principle of justification, any increase of risk must be considered in the light of the substantial benefits of CT and should outweigh the long-term risks [2, 5, 10]. The optimization principle implies that keeping patient radiation doses as low as reasonably achievable (ALARA) should be a priority [1, 10]. It is important to evaluate received dose during CT procedure and estimate the diagnostic reference levels (DRLs) which serve as a basis for the system of optimization and the protection of patients from radiation exposure. DRLs are an important tool for identifying cases where the levels of doses are unusually high. The International Commission on Radiological Protection first introduced the term ‘diagnostic reference level’ in 1996 in Publication 73. DRLs were established further to reduce institutional differences between countries and hospitals. DRLs ensure that the doses delivered to patients, especially children, follow the ALARA principle since children have a higher risk than adults from the detrimental effects of radiation [1, 12]. In 2001, the Commision promoted the use of local DRLs to achieve best practice and obtain optimum range of values for the specific medical imaging protocols. However, since the limited number of studies on paediatric CT doses and DRLs are done, we seek to fill this gap. 

The aim of this study was twofold. Firstly, to estimate the ionizing radiation exposure doses and set local DRLs for head CT examinations according to age. Secondly, to compare local DRLs with national and European DRLs and with literature data in other countries.

## Materials and methods

Scan parameters of single-phase CT examinations of the head performed over a 1-year period were collected from Siemens Somatom Sensation 64 CT scanner. Relationships between dose parameters (effective dose and dose–length product (DLP)) and patients’ age were evaluated. Patients were grouped by age in the following intervals: <1, 1−5 year, 5−10 year, 10−15 year and 15−18 years. Local age-based DRLs, set as the 3^rd^ quartile of the median DLP, were calculated. 

According to European Guidelines on Diagnostic Reference Levels for Paediatric Imaging [12], European DRLs are presented as age-based data for head CT examinations. In order to compare our locally set DRLs with the European DRLs, patients were grouped into the proposed age categories: 0–3 months; 3 months – 1year; 1–6 years; 6–18 years. 

Literature analysis was performed on *PubMed *search engine on inclusion criteria: publication date 2015–2020, used keywords *paediatric computed tomography, paediatric CT, diagnostic reference levels*. The 23 articles discussing paediatric local DRLs were further analyzed.

## Results

Data was collected from 194 paediatric head CT examinations performed during the 2019 year period. The estimated median and mean DLP values for <1, 1−5, 5−10, 10−15 and 15−18 year age groups are shown in Table 1. Estimated median DLP values for routine head CT were 116.1 mGy∙cm for 0−3 months old group, 163.4 mGy∙cm for 3 months − 1 year old group, 231.9 mGy∙cm for 1–6 year old group, 284.2 mGy∙cm for ≥ 6 year old group. Mean DLP values for head CT according to the same age grouping method were 109.4 mGy∙cm for 0−3 months old group, 171.7 mGy∙cm for 3 months − 1 year old group, 245 mGy∙cm for 1–6 year old group, 300.3 mGy∙cm for ≥ 6 year old group. 

**Table 1.** Literature review of paediatric head CT exposure parameters and DRLs.

Country	Age groups	Exposure parameters		Median (*mean*)
		kVp	mAs	DLP, mGy∙cm
Our study	<1 y	100	120 (mean)	**144.3 **(*145.4*)
	1–5 y	100	155	**233.7 **(*249.9*)
	5–10 y	100	177	**246.4 **(*280.2*)
	10–15 y	100	195	**288.9 **(*300.4*)
	15–18 y	100	204	**315.5 **(*344.7*)
Sudan [14]	<1 y	110–120	99–231	*265*
	1–5 y	120–140	104–228	*305*
	5–10 y	120–140	123–241	*407*
Japan [15]	<1 y		120	**398.4**
	1–5 y	80–130	150	**463.5**
	5–10 y		225	**593.6**
India [16]	<1 y	80–120	80–250	*300*
	1–5 y			*304*
Jordan [17]	<1 y			**644.8**
	1–5 y	–	73–217	**874.9**
	5–10 y			**1038.4**
	10–18 y			**1097.5**
Nigeria [18]	<1 y	80–120	73–90	**498**
	1 y	100–120	50–150	**565**
	5 y	100–120	100–160	**782**
	>10 y	100–120	100–180	**889**
Iran [19]	<1 y	120	144 ± 27	**207.2**
	1–5 y	120	144 ± 25	**216.9**
	5–10 y	120	150 ± 17	**232.8**
	10–15 y	120	147 ± 21	**268.6**
Greece [20]	<1 y	100	110–285	
	1–5 y	120	114–300	–
	5–15 y	120	129–350	
Malaysia [21]	<1 y	100	80–380	**250.1**
	1–5 y	100	90–423	**449.0**
	5–10 y	100–120	20–400	**458.5**
	10–15 y	120	80–430	**814.1**

The established local DRLs for head CT (Fig.1) were 170 mGy∙cm for <1 year old group, 300 mGy∙cm for 1–5 year old group, 310 mGy∙cm for 5–10 year old group, 320 mGy∙cm for 10–15 year old group and 360 mGy∙cm for 15−18 year old group. In order to compare local DRLs with European DRLs, we calculated local DRLs according to the different age grouping and they were 130 mGy∙cm for 0−3 months old group, 210 mGy∙cm for 3 months − 1 year old group, 275 mGy∙cm for 1–6 year old group, 320 mGy∙cm ≥ 6 year old group (Table 2).

**Table 2.** Paediatric head CT local DRLs in comparison with national and European DRLs.

CT exam	Category	Number of CT exams	Setted local DRLs	National DRLs [13]	European DRLs [12]
			DLP, mGy∙cm	DLP, mGy∙cm	DLP, mGy∙cm
**Head**	0–1 y	19	170	570	–
	1–5 y	65	300	630	–
	5–10 y	52	310	650	–
	10–15 y	40	320	830	–
	15–18 y	18	360	–	–
	0–3 m	8	130	–	300
	3 m – 1 y	10	210	–	385
	1–6 y	67	275	–	505
	6–18 y	81	320	–	650

## Discussion and conclusions

Health care institutions use different modalities of CT scanners. Data analysis showed that hospitals examine patients using relatively different values of scan parameters (tube voltage (kVp), effective tube current (mAs), scan length (mm), slice thickness (mm) and pitch) which resulted in varying exposure doses for the same CT examinations (Table 1). The range of tube voltage used in other hospitals is between 80 and 140 kVp, whilst effective tube current values range from 20 to 430 mAs. 

The lack of sufficient training of radiology technologists may result in different exposure doses from the same type of radiological procedures performed at different radiology departments. In order to minimize these differences and optimize patient exposure, the assessment of median doses in hospital and comparison with national DRLs plays an important role. National DRLs are set at the 3^rd^ quartile of a wide scale surveys on the median doses obtained from radiology departments in the country for a specific type of examination. They are expected not to be exceeded for standard procedures. Our study results showed that head CT doses were 2–3 times lower than national DRLs and about 2 times lower than European DRLs and indicate that good practice for these procedures is applied in hospitals. However, national DRLs are not optimum doses, they tend to be higher than local DRLs established in hospitals, since they represent the whole country data and practices. National DRLs help to identify potentially unusual practice. Local DRLs are based on the 3^rd^ quartile (the 75^th^ percentile) value of the distribution of patient doses in a hospital intended to act as benchmark levels for doses from common diagnostic procedures. The local DRLs (in terms of DLP) established in this study were compared with data from hospitals of 17 countries based on the same age grouping method (<1, 1−5, 5−10 and 10−15 years) (Fig.1). The results showed that local DRLs for head CT in this study were significantly lower than local DRLs established in other countries.

**Fig. 1.**
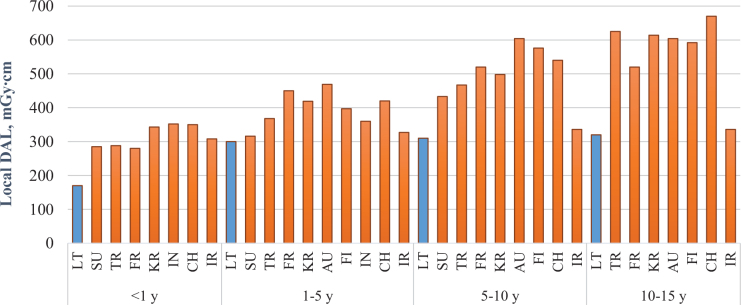
Comparison of paediatric local DRLs for head CT examinations in hospitals.

Note: LT – Lithuania, SU – Sudan, TR – Turkey, FR – France, KR – Korea, AU – Austria, FI – Finland, IN – India, CH – Switzerland, IR – Iran

**Fig. 1 continued:**
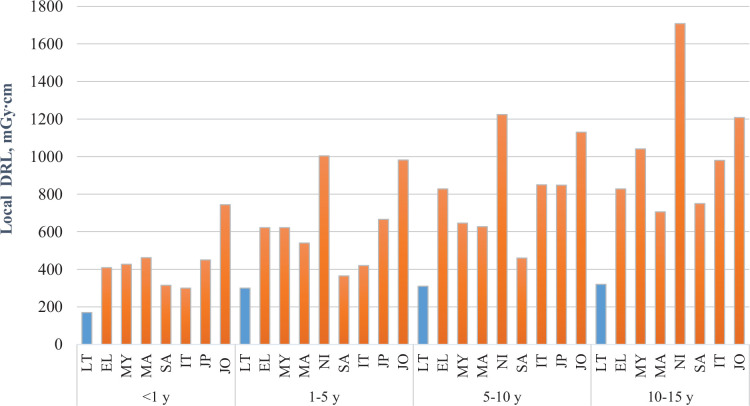
(Comparison of paediatric local DRLs for head CT examinations in hospitals).

Note: LT – Lithuania, EL – Greece, MY – Malaysia, MA – Marocco, NI – Nigeria, SA – PAR, IT – Italy, JP – Japan, JO – Jordan

Despite the importance of reducing patient adverse health effects through dose optimization only limited number of studies on paediatric CT doses were published during last 6 years and none from other hospitals in Lithuania. During comparison of the study results with published data we came to conclusion that mean and median paediatric head CT doses estimated by us were significantly lower (Table 1). Relatively lower estimated doses of head CT procedures in radiology department in 2019 could be explained by the performed exposure optimization studies [22-27]. Patient exposure optimization was done by a long term collaborate work of radiation protection specialists, medical physicists, radiologists, and radiology technologists. Studies included analysis of doses, changes to the acquisition protocol parameters, analysis of image quality done by radiologist and approval of optimized parameters retaining sufficient image quality, setting local DRLs. X-ray source voltage was decreased from 120 kVp to 100 kVp, tube current time product adjusted from 380 mAs to 300 mAs, and an optimal value of 250 mAs was achieved. Between 2010 and 2014, the mean DLP for head CT exams decreased around 50% (from 1690 mGy∙cm to 708 mGy∙cm) [25]. The estimated mean DLP for CT exams in 2019 showed that dose was reduced by additional ~50% for patients of <1 year and ~ 20-25% for those of 1 year and older, from 2014. Griciene et al. [26] showed that children head CT dose decreased in all age groups, from 2010 to 2017, and up to 3 times in the youngest patients. In 2017, the median DLP value for head was 156, 287, 274, 300 and 351 mGy∙cm for patients aged 0–1, 1–5, 5–10, 10–15 and 15–18 years, respectively [26]. Additionally, optimization of head CT protocols for specific indications such us nonsyndromic craniosynostosis was performed at the hospital [27]. The results indicated that CT images acquired with 120 kV and 13 mAs (the lowest mAs setting) were able to achieve similar diagnostic information as with the standard protocol (tube voltage of 120 kVp; automatic tube current modulation 195 mAs) while reducing the patient radiation dose by approximately 94% [27]. Due to the reason that received CT doses using new protocols for patients with nonsyndromic craniosynostosis diagnosis are very low and these patients comprise low percentage in total patient group, they were excluded from our study group. Several limitations exist in this study. First of all, the DRLs of our study were estimated using data collected from a single CT scanner. Also, since paediatric patients were grouped according to age, the sample size for each group fills the requirements for setting DRLs, but is relatively small, especially in youngest age group. The comparative component of our study was limited by a small number of publications on this topic and the lack of international uniformity in age stratification for DRLs. The analysis of other dose metrics such as CTDI_vol_ was not carried out, and there were difficulties in comparing scan parameters of other hospitals since not all countries reported them in their studies. 

Conclusions. The results of our study showed that estimated median doses and settled local DRLs for paediatric head CT were significantly lower than national and European DRLs and published data in other countries. Patient dose assessment and establishment of local DRLs plays important role in future exposure optimization.
